# *Salix* transect of Europe: patterns in the distribution of willow-feeding psyllids (Hemiptera: Psylloidea) from Greece to arctic Norway

**DOI:** 10.3897/BDJ.8.e53788

**Published:** 2020-05-18

**Authors:** Diana Percy, Quentin Cronk

**Affiliations:** 1 University of British Columbia, Vancouver, Canada University of British Columbia Vancouver Canada

**Keywords:** biogeography, ecospace, Europe, Hemiptera, latitudinal gradient, megatransect, oligophagy, Psyllidae, Salicaceae, *Salix* feeders, spatial analysis, Triozidae, willow-feeding insects

## Abstract

**Background**

Psyllids are oligophagous phytophagous insects with many specialist willow (*Salix* spp.) feeding species in two genera (*Cacopsylla* and *Bactericera*). We examine the patterns of distribution and co-occurrence of willow-feeding species at 42 willow sites across Europe forming a transect from Greece (lat. 38.8 °N) to arctic Norway (lat. 70.6 °N). The transect and sites have been described in previous papers.

**New information**

A total of 1245 individual psyllids were examined from 23 species of willow over the transect, representing 17 willow-feeding species (11 *Cacopsylla* and 6 *Bactericera*). Numerous species were very widely distributed, with two species, *Bactericera albiventris* (Foerster, 1848) and *Cacopsylla pulchra* (Zetterstedt, 1840), occurring from Greece to Finland. Other widespread species (Romania to Finland) were *Cacopsylla ambigua* (Foerster, 1848) and *Bactericera curvatinervis* (Foerster, 1848). The mean number of psyllid species per site was 2.4 (1.3 *Cacopsylla*, 1.1 *Bactericera*).

## Introduction

The megatransect of European lowland willow sites has already been described (Cronk et al. 2015). A total of 42 sites (with some supplementary sites) were sampled for willow associated chrysomelid beetles (Coleoptera: Chrysomelidae; Canty et al. 2016, Canty et al. 2019) and weevils (Coleoptera: Curculionoidea; Canty et al. 2020), and salicivorous psyllids (this paper). In addition, nettles (*Urtica dioica* L.) at the sites were assessed for ploidy level (Cronk et al. 2016) and the associated nettle-feeding psyllid (*Trioza urticae* (Linné, 1758)), was collected for phylogeographic analysis (Wonglersak et al. 2017).

Psyllids, or jumping plant lice, are members of the hemipteran superfamily Psylloidea (Hodkinson 2009, Hodkinson 1974) with eight families (Burckhardt and Ouvrard 2012, Burckhardt 2011). They are inconspicuous phloem-feeding insects showing pronounced oligophagy, usually with a host range comprising a single plant species or a group of related species (Ouvrard 2019, Ouvrard et al. 2015). Two psyllid genera have independently evolved clades of species using willows as primary hosts: *Bactericera* (Triozidae) and *Cacopsylla* (Psyllidae) (Percy et al. 2018).

**Figure 1.**
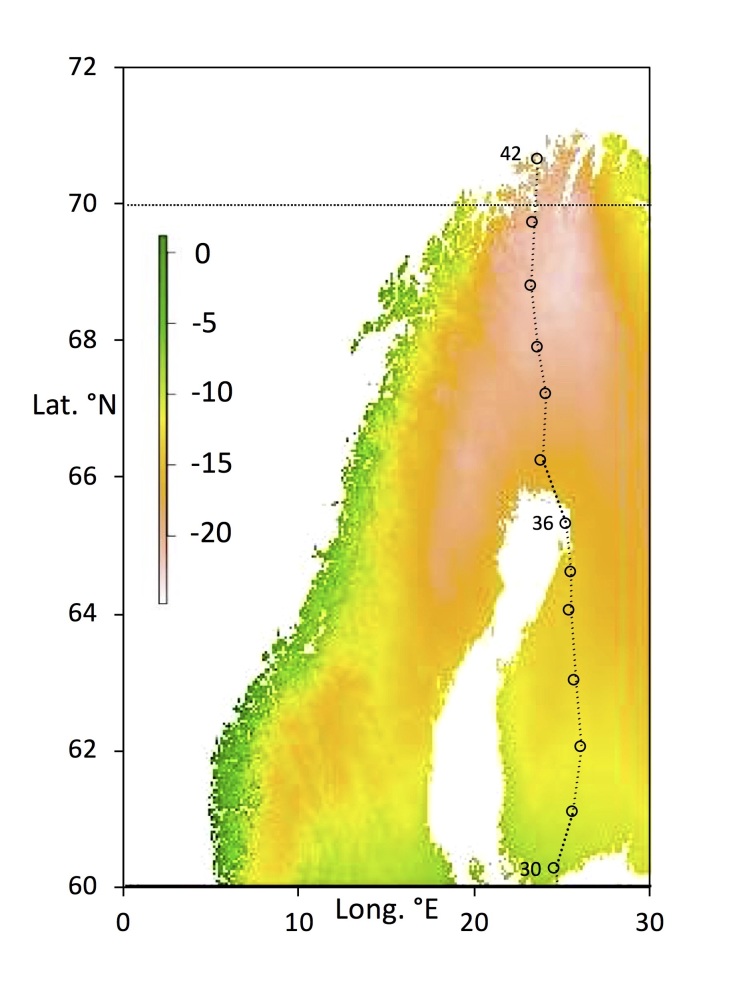
Northern sites (numbers 30 – 42; Lat. 60.27°N–70.65°N), showing the distribution of extreme low winter temperatures in Fennoscandia, as mean minimum monthly temperature for January (scale in °Celsius).

**Figure 2.**
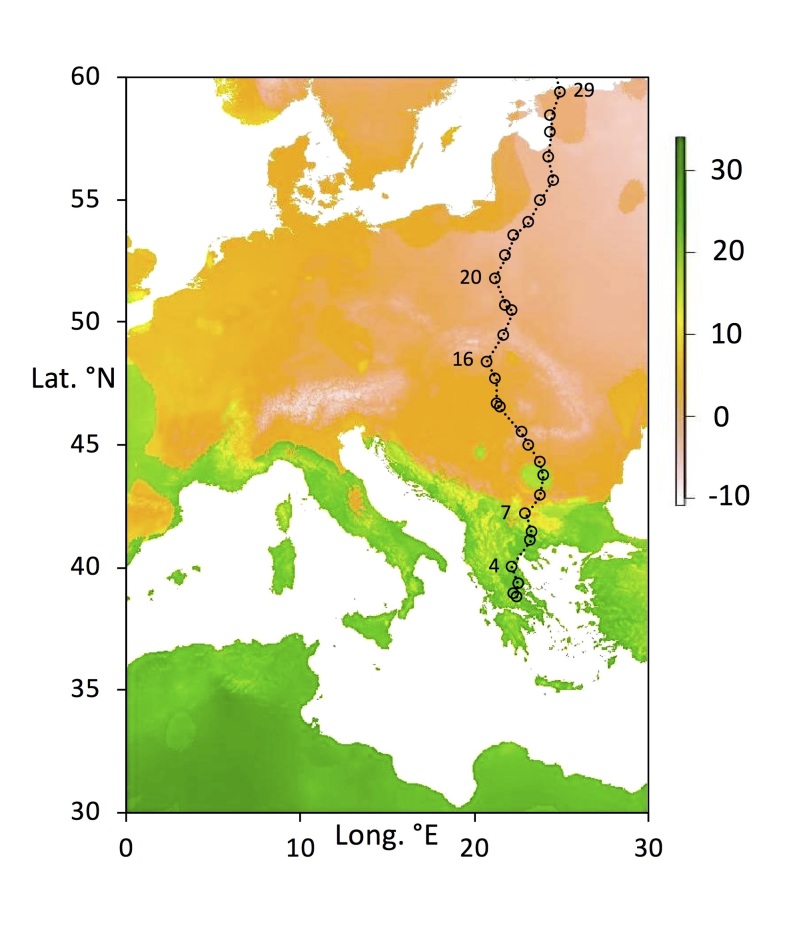
Southern sites (numbers 1 – 29; Lat. 33.80°N–59.40°N), showing the mean temperature of the driest quarter (scale in °Celsius). This parameter clearly shows the boundary of the hot and dry summer Mediterranean region (green) as opposed to winter-dry central Europe. Bioclimatic parameter (Bio9) extracted from WorldClim.

The megatransect used here encompasses a wide variety of climatic conditions. A major transition is between the summer dry Mediterranean and the winter-dry central European plain (Figure 1). At the far north of Fennoscandia extreme winter temperatures prevail (Fig. 2). It is therefore of interest to determine to what extent willow psyllids tolerate widely varying climates in order to achieve wide distributions.

Temperature and water availability are major drivers of psyllid life history variation (Hodkinson 2009). Temperature is critically important to control the development of immatures, with different species having different optima. Developmental rates of the Australian psyllid, *Boreioglycaspis melaleucae* Moore, 1964 (used as a biocontrol of *Melaleuca quinquenervia* in Florida) increased linearly with increasing temperature to an optimum 25°C (Chiarelli et al. 2011). In contrast, temperature in May, and growing season above 3°C were found to be significant in determining the distribution of the northern hemisphere psyllid *Strophingia ericae* (Curtis, 1835) (Hodkinson et al. 1999). In addition, *S. ericae* has developmental plasticity to adapt to low temperature environments that slow down development by switching from a 1-year life cycle to a 2-year life cycle; while other taxa exhibit univoltine or multivoltine life cycles depending on warmer or cooler regions over the species distribution, or across seasons depending on varying ambient temperatures (Hodkinson 2009). In *S. ericae* the rate of development could be completed at 10°C, although it was considerably faster at 15°C (Miles et al. 1998). On the other hand, in *Diaphorina citri* Kuwayama, 1908, the subtropical citrus psyllid, immatures fail to complete development at 15°C (Nakata 2006). In arctic Alaska, the temperature for development has been suggested as critical for determining the distributional envelope of psyllid species, which are often more restricted than that of the willow hosts (MacLean 1983). Although immature development is largely determined by temperature, photoperiod may be important in entering developmental quiescence. Experiments on *Strophingia* have shown that while development of immatures in the spring is temperature regulated, developmental inhibition in autumn, to enter winter quiescence, is determined by short photoperiod (Miles et al. 1998).

Despite the evidence for critical temperatures in development, psyllids nevertheless seem to be generally tolerant of extreme low temperatures, and absolute low temperatures are rarely implicated in determining psyllid distributions. The Ericaceae-feeding psyllids, *Strophingia*, are low temperature tolerant at least down to -15°C (Hodkinson et al. 1999). Even the subtropical citrus psyllid, *D. citri*, displays considerable tolerance of sub-zero temperatures (Hall et al. 2011). Oviposition thresholds in this species are 16-41.6°C with an optimum at 29.6°C (Hall et al. 2011), so tolerance of sub-zero temperatures might seem surprising. Similarly, extreme high temperatures rarely seem to determine psyllid distribution, as high temperatures are mitigated by evaporative cooling from the plant host (Hoffmann et al. 1975). Nevertheless, high summer temperatures have been implicated as a limiting factor in outbreaks of the potato psyllid, *Bactericera cockerelli* (Šulc, 1909) in the American south-west (List 1939); and physiological limitations can be more pronounced and range restrictive amongst co-occurring species (Hodkinson et al. 1999)

A study of willow psyllids in relation to altitude in Norway found evidence of climatic optima, with *Cacopsylla palmeni* (Löw, 1882) and *C. brunneipennis* (Edwards, 1896) at higher and lower altitudes respectively (Hill and Hodkinson 1995). Both species develop only on female catkins and are thus phenologically linked to catkin development. *Salix* feeding psyllids vary as to whether they develop on catkins or leaves, and this has phenological consequences as catkins usually develop precociously, ahead of leaves (Hill et al. 1998). The catkin is a sheltered, albeit temporally restricted, environment for immature development, and adaptation to catkin feeding is a key shift in willow psyllid biology. Male catkins are more ephemeral and so the relatively more persistent female catkins are preferred for oviposition. The association with catkins may have negative consequences for the host. A study in Arctic Alaska showed that densities of immatures in female catkins can be extremely high and negatively affect catkin growth (Hodkinson et al. 1979)

Individual species of willow psyllid may oviposit and develop on several related species of willow. For instance, *Cacopsylla groenlandica* (Šulc, 1913) in Greenland (Hodkinson 1997) makes use of *Salix glauca*, *S. arctophila*, *S. uva‐ursi* and *S. herbacea*. However, at the northern range limit *C. groenlandica* only developes on the female catkins of *S. glauca*. Under favourable environmental conditions the use of multiple host species may allow ecological expansion in time and space.

Our study, using single season sampling over a large latitudinal range provides a “snap shot” of distribution and abundance at each site with variable climate-host compositions. This lays a baseline that long term repeat sampling can refer to, to assess changes in composition of willows and willow associated insects as the environment of the transect changes. Here we present data for the willow-feeding psyllids to complement data already published for willows and beetles.

## Material and methods

### Collection methods

The 42 willow sites (Figs 1, 2) for collecting were selected as described previously (Cronk et al. 2015). Basic site details are given in Table 1, with further details in Cronk et al. 2015). The sites form a “megatransect” from Greece (lat. 38.8°N) to arctic Norway (lat. 69.7°N) along roughly the same line of longitude (Table 1). Psyllids were collected from willows (*Salix* spp.) by DP by sweep netting for c. 1 hour at each site (see Canty et al. 2016 for further details of insect collecting at the sites). Psyllids were collected into 95% alcohol and held at room temperature until transferred to long term storage at -20°C at the University of British Columbia (UBC) for analysis. Voucher specimens of all taxa have been deposited at the Beaty Biodiversity Museum, UBC (Vancouver, Canada).

### Specimen preparation, examination and identification

Specimens in ethanol were subjected to preliminary sorting, followed by clearing of 2 to 5 specimens of each species per site in KOH (10 mins), and subsequent dehydration by alcohol series to return them to 95% ethanol for inspection of cleared material. Cleared specimens were examined under a stereomicroscope at magnifications of up to x50. Species were identified using regional faunas, primarily Ossiannilsson 1992, Hodkinson and White 1979.

### Climate

Climate variables from WorldClim (Hijmans et al. 2005; http://www.worldclim.org), interpolated on a 30 arc-second (~1km) grid, as monthly means (1950-2000), extracted using the data portal at the Senckenberg Biodiversity and Climate Research Centre in Frankfurt (http://dataportal-senckenberg.de/dataExtractTool). Climate is shown graphically by means of the hythergraph: a plot of monthly precipitation (mm) against mean monthly temperature (°C). Whereas a climograph is any graphical representation of climate, a hythergraph specifically refers to a plot of temperature against precipitation, as coined by T. Griffith Taylor (Taylor 1918). As precipitation often varies much more than temperature, a log scale is used here for the former. We extend the hythergraph by plotting lines of equal effective pluviality (pluv = rainfall (mm)/(25+t°C)^2^ x 0.0018) based on the Ivanov formula for evapotranspiration (Molle et al. 1999). These lines give a simple temperature correction for the effectiveness of precipitation, and they are a measure of equivalent wetness of the climate from precipitation over different temperatures.

### Data Analysis

The association between psyllid occurrences and latitude were analysed using canonical correspondence analysis (CCA). The psyllid occurrence matrix (presence and absence of species) was used as the response matrix and latitude as the explanatory matrix. Site 9 (no psyllids) was omitted, as were species found at only one site. For similarity decay with distance (SDD) analyses ([Bibr B5759925][Bibr B5760065]), similarity (S) of psyllid fauna between sites was measured using the Jaccard similarity coefficient (with conversion into distance (D) as D=1-S). Jaccard similarity was used as this is a widely used and robust measure that does not overemphasize shared distances. Multivariate analysis and calculation of distance/similarity matrices was carried out using the Java package Ginkgo in the software suite B-VegAna (Bouxin 2005, Font Castell 2007). Geographical distance between sites was calculated using GDMG (Ersts 2012).

## Results

### General patterns of psyllid occurrence

The direct geographical distance from site 1 (Greece) to site 42 (Norway) was 3247 km. Table 2 details the total of 17 willow-feeding species that were recorded (11 *Cacopsylla* and 6 *Bactericera*). Numbers of species per site varied from 0 (site 9, Bulgaria) to 6 (site 28, Estonia). All other sites had between 1 and 4 species. The mean number of psyllid species per site was 2.4 (1.35 *Cacopsylla*, 1.05 *Bactericera*).

**Figure 3.**
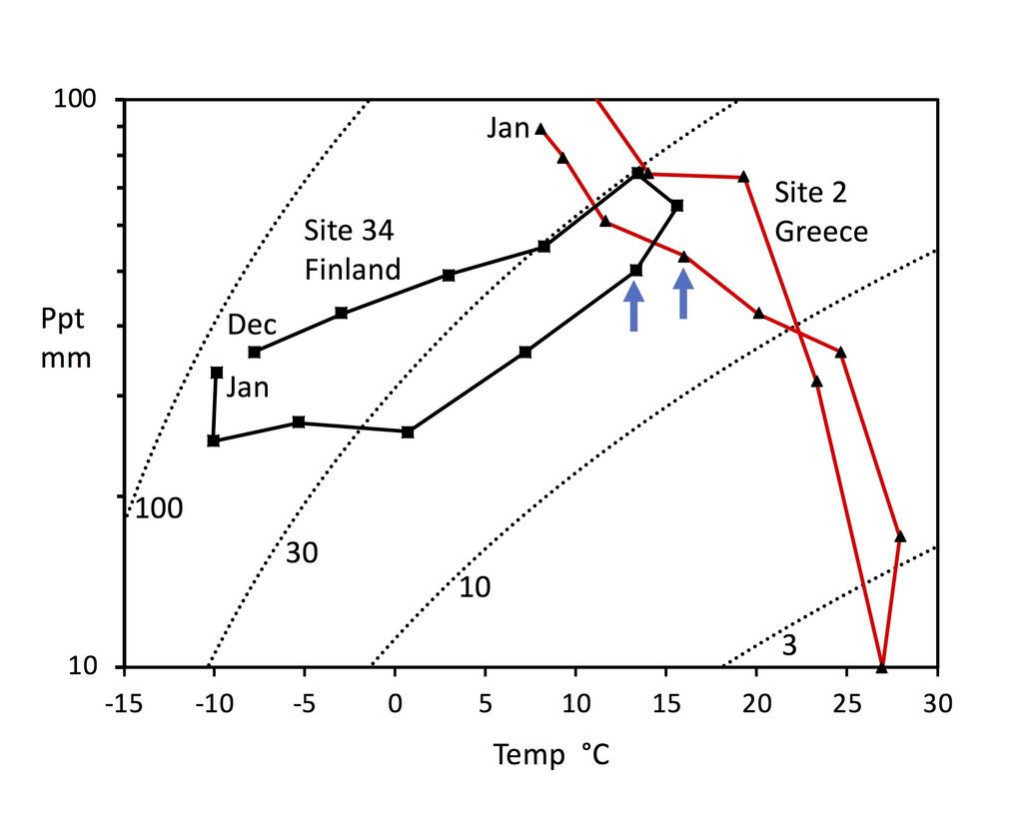
Comparison of the climates at the northernmost and southernmost localities for *Bactericera albiventris*. Hythergraph showing mean monthly temperature and precipitation (see methods for details). The climate track for the Finnish site shows the winter-dry, summer-wet climate, whereas the climate track for the Greek site shows the winter-wet, summer-dry climate characteristic of the Mediterranean. Collection months are arrowed.

Four species occurred in 10 or more sites: *Cacopsylla saliceti* (Foerster, 1848) (17 sites: mainly southern), *Cacopsylla pulchra* (Zetterstedt, 1840)(13 sites: widespread), *Bactericera striola* (Flor, 1861) (10 sites: throughout Finland) and *Bactericera albiventris* (Foerster, 1848) (20 sites: widespread). The species with the widest geographical distribution were *B. albiventris* and *C. pulchra*, both occurring from Greece to Finland. Fig. 3 shows a representation of the climate at the southernmost and northernmost sites for *B. albiventris* (sites 2 and 34), Note that the summer climate of Finland is very similar to the spring climate of Greece.

Other widespread species (Romania to Finland) were *Cacopsylla ambigua* (Foerster, 1848) and *Bactericera curvatinervis* (Foerster, 1848). Three taxa, found only at single sites, remain unidentified: *Cacopsylla* sp. [S6H6] (site 6, Bulgaria), *Cacopsylla* sp. [S17H2] (site 17, Poland), *Bactericera* sp. [S21H4] (site 21, Poland). These are likely described species with insufficient material to determine, but may represent undescribed species. *Cacopsylla brunneipennis* appears to be a new record for Hungary and is not included in Ripka 2008, Ripka 2010. Not all expected European willow feeding psyllids (Table 3) were found in our samples. For instance, *Bactericera versicolor* (Löw, 1888) and *Cacopsylla parvipennis* (Löw, 1878), although known from central Europe, are not recorded here.

### Quantitative association with latitude

**Figure 4.**
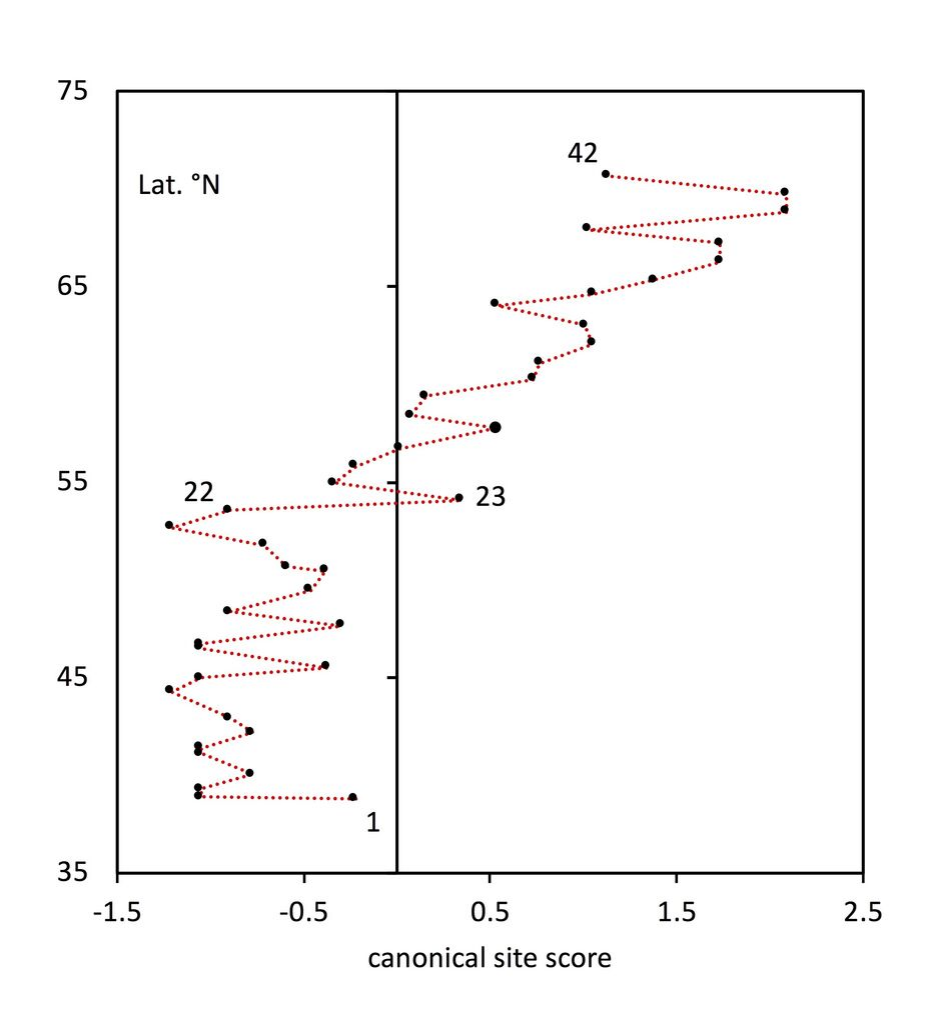
Comparison of site latitude with site scores on the latitude-constrained CCA axis. Correlation between the two would indicate that species composition at sites is strongly associated with latitude. The southern sites show no strong latitudinal pattern (sites 1-22: R² = 0.0165) whereas northern sites do (sites 23-42: R² = 0.756).

The canonical correspondence analysis (CCA) gave a single canonical axis reflecting the variation in the data matrix that is best explained by latitude. The canonical axis (latitude) explains 19.15% of the variation, while the first non-canonical axis explains 21.16%. When the first canonical axis is then compared with latitude (Fig. 4) it can be seen that the association of species composition with latitude is mainly due to the sites above 23 (northern Poland) which show a general trend of increasing CCA score with latitude (sites 23-42: R² = 0.756). This indicates that there is a strong latitudinal trend in northern Europe (Baltic and Fennoscandian region) but a relatively homogeneous psyllid fauna south of that (Greece to southern Poland) with little latitudinal trend (sites 1-22: R² = 0.0165).

### Patterns of Host Association

Multiple psyllids were found on most of the willow species (Table 4) with the exception of *S. amplexicaulis*, *S. euxina* and *S. gmelinii*, which had only one psyllid species recorded from each. However, these were relatively uncommon willows on our transect and further sampling might have revealed other psyllid species. Similarly, no psyllids were confined to a single willow host, but there were clear patterns of preference, where this could be determined reliably in the commoner psyllids, i.e. those found at five or more sites (Table 5). For instance, *B. albiventris* and *C. saliceti* have a strong association with *Salix alba*, whereas *B. striola* has a very strong association with *S. phylicifolia*. Where psyllid occurrence is only marked by a plus sign (+) in Table 5, the occurrence may be only casual, the willow only being used for resting and/or feeding, but not necessarily breeding. In a few cases it was possible to confirm a breeding association by the collection and identification of immatures. These cases are indicated by asterisks in Table 5.

Of the rarer psyllids (<5 sites) the host occurrences were as follows: *Bactericera salicivora* (Reuter, 1876) (*S. myrsinifolia*), *Bactericera* sp. [S21H4] (*S. viminalis*, *S. x fragilis*), *Cacopsylla moscovita* (Andrianova, 1948) (*S. viminalis*, *S.* x *fragilis*, *S. myrsinifolia*, *S. cinerea* x *S. aurita*, *S. caprea*, *S. bebbiana*), *Cacopsylla nigrita* (Zetterstedt, 1828)(*S. phylicifolia*, *S. glauca*), *Cacopsylla propinqua* (Schaefer, 1949)(*S. glauca*, *S. gmelinii*), *Cacopsylla* sp. [S17H2] (*S. purpurea*), *Cacopsylla* sp. [S6H6] (*S. alba*, *S.* x *fragilis*), *Cacopsylla zaicevi* (Šulc, 1915) (*S. glauca*, *S. hastata*). These less common psyllids were generally collected on one or two willow species only. An exception was *C. moscovita*, which although found only at three sites, these sites were willow-rich and *C. moscovita* was found widely on the willow species present.

There is some indication of a *Salix* taxonomic signal in the host preferences of psyllids. For instance, *Bactericera albiventris* is found commonly on *S. triandra*, *S. alba*, *S.* x *fragilis* (all subgenus Salix) and rarely on other willows (subgenus Vetrix). In contrast, *Cacopsylla pulchra* is found commonly on *S. purpurea*, *S. myrsinifolia*, *S. cinerea*, *S. cinerea* x *aurita* (all subgenus Vetrix) and rarely on subgenus Salix. However, there is no indication of a systematic difference between *Bactericera* and *Cacopsylla* in host choice, as species of both genera occur widely on a variety of hosts.

### Species turnover along the transect

**Figure 5.**
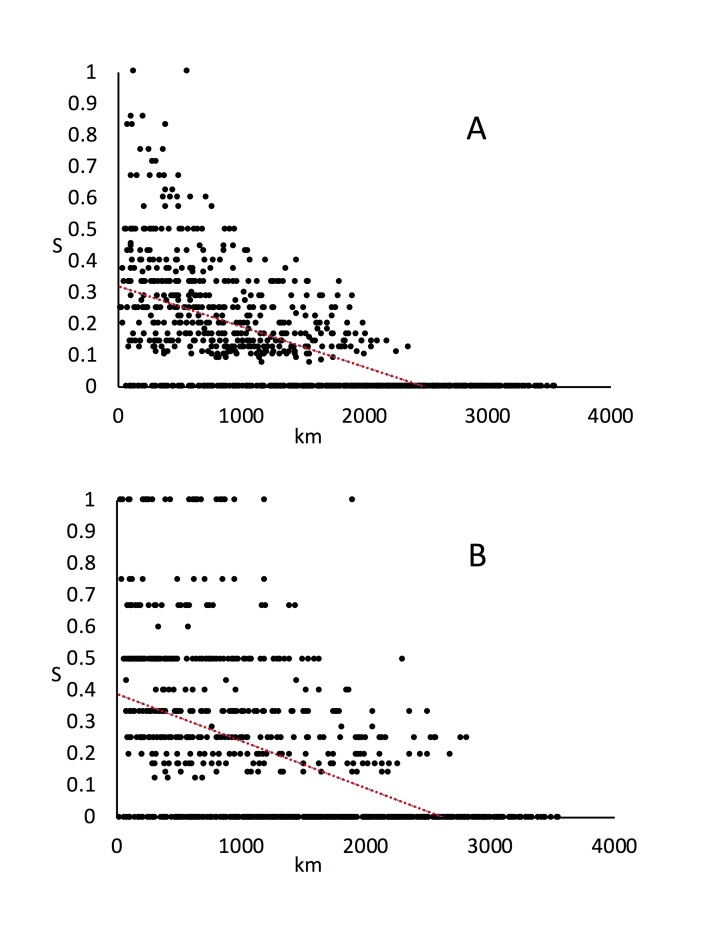
Similarity decay with distance (SDD) analysis. Plot of decreasing site similarity (Jaccard similarity coefficient, based on: A = willows; B = psyllids) with geographical distance (km). The red trendline shows the linear regression. The patterns show similar gross geographical patterning between willows and psyllids.

We used similarity decay with distance (SDD) analysis (Nekola and White 1999, Steinitz et al. 2006) to investigate the scale of geographical patterning in willows and psyllids. Fig. 5 shows the plots of Jaccard similarity against distance. The slope of the regression line and the values of the intercepts on the x and y axes are given in Table 6. The patterns are broadly similar for psyllids and willows. The x-intercept, in kilometres, gives a measure of the approximate distance needed (in this case in a north-south direction) to reach a completely different fauna or flora (i.e. a similarity of zero). In other words, the distance taken for one biota to be replaced geographically by another. The y-intercept gives a measure of the similarity (S_Jaccard_) of communities in a local area (i.e. when km = 0). This (or rather 1-S) is an index of local community diversity. By this measure willow communities have somewhat more local variation than psyllids (0.6797 vs 0.6103) but overall the results are similar. The similarity decay distances of 2633 km vs 2502 km, for psyllids and willows respectively, are remarkably similar.

## Discussion

It is clear from previous studies of psyllid biology that there is tight ecological integration between individual psyllid species and their hosts, for instance in phenological synchronicity, and in feeding choice using particular elements of willow morphological space such as catkins (Hodkinson 1997, Hodkinson 2009). This paper investigates whether this integration also extends to the macroecological realm by studying psyllid and willow distribution on a trans-continental scale. Many willow species are known to have very wide distributions, with well-known species such as *Salix alba* (the white willow) extending over much of Europe. Psyllids match this pattern with many very widespread species. Our data provide quantitative support for such a geographical match based on comparing results of a similarity decay with distance analysis.

We show that the psyllid fauna varies across Europe, but largely in response to increasing boreality in the north. The enormous climatic difference between the Mediterranean region and the central European plain seems (from our data) to make little difference to the psyllid fauna.

We also provide evidence that there is broad-scale patterning of host use, particularly with regard to subgenus Salix vs subgenus Vetrix. Although individual psyllid species are clearly able to utilize numerous related willow hosts depending on what species are available, there does seem to be a distinct division between Vetrix specialists and Salix specialists. Willows are taxonomically complex with many recorded hybrids (Percy et al. 2014), and the occurrence of psyllids on multiple willow species and hybrids may be facilitated by hybridization in willows (e.g., the hybrid bridge hypothesis; Floate and Whitham 1993).

This study provides a baseline to use in future analyses of geographical shifts and responses to climate. In addition, sampling more (both temporally and geographically) sites and habitats will undoubtedly yield more diversity (e.g. psyllids on alpine willows). Hodkinson and Bird (1998) note that herbivorous insects, and in particular salicivorous psyllids, could act as "biosensors" due to the capacity to respond rapidly to changes in mean temperature resulting from climate shifts. Currently, the scale of sampling on the *Salix* transect provides a baseline and observations will require follow on sampling. For instance, our data finds that more species have a northern median distribution, and some species records are new, e.g. *Cacopsylla ambigua* and *C. abdominalis* were not found south of Romania or Lithuania, respectively, in our sampling, but both have previously been reported for Greece (Ouvrard 2019); conversely, in Greece we found *C. saliceti* and *C. pulchra*, neither of which have been recorded there previously (although both are widespread and known from Bulgaria and Italy). However, clearly these findings need to be augmented with local surveys.

Despite the obvious limitations of a rapid survey megatransect approach, there are increasingly sophisticated ecological meta-analysis approaches that provide opportunities to combine large and local scale surveys at independent data scales in order to address big science questions (e.g. Mammola et al. 2019, Westgate et al. 2014).

## Conclusions

A rapid survey transect of the willow-feeding psyllids of Europe has provided a "snapshot" of the diversity of salicivorous psyllids on a continental scale. At 42 sites across Europe along a latitudinal gradient, we collected 1245 psyllids from 23 species of willow, representing 17 willow-feeding species (11 *Cacopsylla* and 6 *Bactericera*). Patterns of distribution and host association were evident. Numerous species were very widely distributed, with two species, *Bactericera albiventris* (Foerster, 1848) and *Cacopsylla pulchra* (Zetterstedt, 1840), occurring from Greece to Finland.

## Figures and Tables

**Table 1.** Basic site details and numbers of species of psyllid collected. See Cronk et al. 2015 for further details.

**SITE no.**	**Country**	**Lat. °N**	**Long. °E**	**Alt (m)**	**Date of collection (2015)**	***Cacopsylla* (no. of spp.)**	***Bactericera* (no. of spp.)**	**Total spp.**
1	Greece	38.80007	22.46290	37	21 April	1	0	**1**
2	Greece	38.90200	22.31015	33	21 April	1	1	**2**
3	Greece	39.30669	22.52832	177	22 April	1	1	**2**
4	Greece	40.03268	22.17544	534	22 April	2	1	**3**
5	Greece	41.11332	23.27389	31	23 April	1	1	**2**
6	Bulgaria	41.41247	23.31861	90	23 April	2	1	**3**
7	Bulgaria	42.16562	22.99814	392	24 April	2	1	**3**
8	Bulgaria	42.92399	23.81056	339	24 April	0	1	**1**
9	Bulgaria	43.73934	23.96675	35	24 April	0	0	**0**
10	Romania	44.26034	23.78678	81	25 April	1	0	**1**
11	Romania	44.96198	23.19034	172	25 April	1	1	**2**
12	Romania	45.51068	22.73722	556	26 April	2	2	**4**
13	Romania	46.51850	21.51284	102	26 April	1	1	**2**
14	Hungary	46.70074	21.31268	94	27 April	1	1	**2**
15	Hungary	47.66565	21.26177	91	27 April	3	1	**4**
16	Hungary	48.37429	20.72526	148	28 April	0	1	**1**
17	Poland	49.46345	21.69725	385	28 April	1	2	**3**
18	Poland	50.47023	22.23837	157	29 April	1	1	**2**
19	Poland	50.67399	21.82339	141	29 April	2	2	**4**
20	Poland	51.77504	21.19710	101	30 April	1	2	**3**
20a	Poland	51.77504	21.19710	101	11 June	1	0	**1**
21	Poland	52.69398	21.85290	96	12 June	1	1	**2**
22	Poland	53.55483	22.30299	128	12 June	0	1	**1**
23	Poland	54.06943	23.11745	137	13 June	2	1	**3**
24	Lithuania	54.92583	23.77420	28	13 June	2	0	**2**
25	Lithuania	55.79557	24.56678	62	13 June	1	0	**1**
26	Latvia	56.71141	24.25162	23	14 June	3	1	**4**
27	Latvia	57.74963	24.40230	7	14 June	3	1	**4**
28	Estonia	58.42257	24.44063	18	15 June	4	2	**6**
29	Estonia	59.40289	24.93577	48	15 June	2	0	**2**
30	Finland	60.27299	24.65843	33	16 June	3	1	**4**
31	Finland	61.09965	25.62820	84	16 June	2	1	**3**
32	Finland	62.04962	26.12369	174	17 June	2	1	**3**
33	Finland	63.01589	25.80457	139	17 June	1	2	**3**
34	Finland	64.05074	25.52664	91	17 June	1	2	**3**
35	Finland	64.61287	25.53805	58	18 June	2	1	**3**
36	Finland	65.32835	25.29175	1	18 June	0	1	**1**
37	Finland	66.24947	23.89450	51	19 June	0	2	**2**
38	Finland	67.21253	24.12629	160	19 June	0	2	**2**
39	Finland	67.91183	23.63411	233	19 June	0	2	**2**
40	Norway	68.81380	23.26658	374	20 June	1	1	**2**
41	Norway	69.72487	23.40581	289	20 June	1	1	**2**
42	Norway	70.65234	23.66583	67	21 June	2	0	**2**
					***MEAN***	***1.35***	***1.05***	***2.4***

**Table 2.** Psyllid species (*Cacopsylla*11 spp.; *Bactericera*, 6 spp.) collected during this study with distributions (sites and countries). For sites refer to Table 1; country abbreviations: Gr (Greece), Bu (Bulgaria), Ro (Romania), Hu (Hungary), Po (Poland), La (Latvia), Li (Lithuania), Es (Estonia), Fi (Finland), No (Norway). Median site: the central tendency of the species distribution is given as site median (low numbers indicate southern species, high numbers indicate northern species), and on the basis of the site distribution, species are classified as southern (S), middle (M), northern (N) or wide (W).

**Sp. no.**	**Species**	**Site numbers**	**Countries**	**No. of sites (tot.)**	**Number of individuals (total)**	**Median site**
1	*Cacopsylla saliceti* (Foerster, 1848)	2 – 7, 10 – 15, 19, 20, 20a, 21, 24	Gr, Bu, Ro, Hu, Po, Li	17	224	12 (S)
2	*Cacopsylla moscovita* (Andrianova, 1948)	23, 27, 28	Po, La, Es	3	22	27 (M)
3	*Cacopsylla propinqua* (Schaefer, 1949)	42	No	1	38	42 (N)
4	*Cacopsylla* sp. [S6H6]	6	Bu	1	2	6 (S)
5	*Cacopsylla pulchra* (Zetterstedt, 1840)	1, 4, 7, 15, 18, 19, 25 – 31	Gr, Bu, Hu, Po, Li, La, Es, Fi	13	>198	25 (W)
6	*Cacopsylla* sp. [S17H2]	17	Po	1	1	17 (M)
7	*Cacopsylla brunneipennis* (Edwards, 1896)	15, 30 – 32, 34, 35, 42	Hu, Fi, No	7	274	32 (N)
8	*Cacopsylla zaicevi* (Šulc, 1915)	41	No	1	6	41 (N)
9	*Cacopsylla ambigua* (Foerster, 1848)	12, 23, 26, 28, 30, 32, 33, 35	Ro, Po, La, Es, Fi	8	118	29 (W)
10	*Cacopsylla abdominalis* (Meyer-Dür, 1871)	24, 26 – 29	Li, La, Es	5	32	27 (M)
11	*Cacopsylla nigrita* (Zetterstedt, 1828)	40	No	1	2	40 (N)
12	*Bactericera striola* Ossiannilsson, 1992	27, 30 – 38	La, Fi	10	73	33.5 (N)
13	*Bactericera curvatinervis* (Foerster, 1848)	12, 17 – 20, 23, 28, 39	Ro, Po, Es, Fi	8	26	19.5 (W)
14	*Bactericera cf. parastriola* Conci, Ossiannilsson & Tamanini, 1988	37 – 41	Fi, No	5	96	39 (N)
15	*Bactericera* sp. [S21H4]	21	Po	1	4	21 (M)
16	*Bactericera salicivora* (Reuter, 1876)	33	Fi	1	1	33 (N)
17	*Bactericera albiventris* (Foerster, 1848)	2 – 8, 11 – 17, 19, 20, 22, 26, 28, 34	Gr, Bu, Ro, Hu, Po, La, Es, Fi	20	128	13.5 (W)

**Table 3.** European species of willow-feeding psyllid; * = present

**Species**	**Europe only**	**Europe and other palaearctic**	**Europe, other palaearctic, nearctic**	**In transect**
*Bactericera albiventris* (Foerster, 1848)		*		*
*Bactericera curvatinervis* (Foerster, 1848)		*		*
*Bactericera maura* (Foerster, 1848)		*		
*Bactericera parastriola* Conci, Ossiannilsson & Tamanini, 1988	*			*
*Bactericera salicivora* (Reuter, 1876)			*	*
*Bactericera salictaria* (Loginova, 1964)		*		
*Bactericera silvarnis* (Hodkinson, 1974)		*		
*Bactericera striola* (Flor, 1861)		*		*
*Bactericera substriola* Ossiannilsson, 1992	*			
*Bactericera versicolor* (Löw, 1888)	*			
*Cacopsylla abdominalis* (Meyer-Dür, 1871)		*		*
*Cacopsylla ambigua* (Foerster, 1848)		*		*
*Cacopsylla atlantica* (Loginova, 1976)	*			
*Cacopsylla brunneipennis* (Edwards, 1896)		*		*
*Cacopsylla elegantula* (Zetterstedt, 1840)		*		
*Cacopsylla flori* (Puton, 1871)		*		
*Cacopsylla intermedia* (Löw, 1888)		*		
*Cacopsylla iteophila* (Löw, 1876)	*			
*Cacopsylla moscovita* (Andrianova, 1948)		*		*
*Cacopsylla nigrita* (Zetterstedt, 1828)		*		*
*Cacopsylla palmeni* (Löw, 1882)			*	
*Cacopsylla parvipennis* (Löw, 1877)		*		
*Cacopsylla perrieri* Lauterer & Burckhardt, 1997	*			
*Cacopsylla propinqua* (Schaefer, 1949)		*		*
*Cacopsylla pulchra* (Zetterstedt, 1840)		*		*
*Cacopsylla saliceti* (Foerster, 1848)		*		*
*Cacopsylla tatrica* Lauterer & Burckhardt, 1994	*			
*Cacopsylla zaicevi* (Šulc, 1915)			*	*
**Total**	**7**	**18**	**3**	**14**

**Table 4.** Classification of psyllid-hosting willows on the transect, with the number of psyllid species recorded in this study, and the number of sites at which the willows were found. The willow classification is taken from Skvortsov (1999); further details of the willow species may be found in Cronk et al. (2015).

** Salix **	**No. of psyllid species**	**No. of sites**	*** Salix *** **subgenus**	***Salix* section**
*S. glauca*	4	5	Chamaetia	Glaucae
*S. triandra*	4	15	Salix	Amygdalinae
*S. triandra* x *viminalis*	2	3	Salix	Amygdalinae/ Vimen
*S. alba*	3	20	Salix	Salix
*S. euxina*	1	4	Salix	Salix
*S.* x *fragilis*	7	13	Salix	Salix
*S. phylicifolia*	7	14	Vetrix	Arbuscella
*S. hastata*	3	5	Vetrix	Hastatae
*S. amplexicaulis*	1	4	Vetrix	Helix
*S. purpurea*	4	8	Vetrix	Helix
*S. purpurea* x *viminalis*	3	8	Vetrix	Helix/Vimen
*S. myrsinifolia*	7	13	Vetrix	Nigricantes
*S. aurita*	3	6	Vetrix	Vetrix
*S. bebbiana* (*S. starkeana*)	4	7	Vetrix	Vetrix
*S. caprea*	4	14	Vetrix	Vetrix
*S. cinerea*	4	9	Vetrix	Vetrix
*S. cinerea* x *aurita*	4	1	Vetrix	Vetrix
*S. silesiaca*	2	1	Vetrix	Vetrix
*S. lapponum*	3	4	Vetrix	Villosae
*S. gmelinii*	1	1	Vetrix	Vimen
*S. viminalis*	7	9	Vetrix	Vimen

**Table 5.** Host associations of psyllids occurring at five or more sites. The host association index is calculated as consistency of association (the number of sites where a psyllid occurs on a particular willow as a percentage of total sites for that psyllid) multiplied by strength of association (the percentage of individuals, from all sites, recorded from that willow). When a psyllid is recorded very occasionally on a particular willow (host association index <1), or the total number of insects for that willow is <5, the association is merely recorded as +. The strongest associations between a psyllid species and a particular willow are marked in bold. In rare cases where host association could be confirmed by immature identifications, this is marked by a double asterisk (**, multiple sites) or single asterisk (*, single site).

** Salix **	***C. pulchra***	***B. albi-ventris***	***C. saliceti***	***B. striola***	***B. curvati-nervis***	***C. ambig-ua***	***C. brunnei-pennis***	***B. cf. para-striola***	***C. abdom-inalis***
*S. glauca*	-	-	-	-	-	-	-	+	-
*S. triandra*	+	2.4	+	-	-	1.3	-	-	-
*S. triandra* x *viminalis*	-	+	+	-	-	-	-	-	-
*S. alba*	-	**27.1**	**25.5**	-	-	-	-	-	-
*S. euxina*	-	+	-	-	-	-	-	-	-
*S.* x *fragilis*	+	7.8	2.4	-	-	-	-	-	+
*S. phylicifolia*	+	+	-	**61.4**	-	+	14.0**	**28.3**	-
*S. hastata*	-	-	-	+	+	-	-	1.4	-
*S. amplexicaulis*	+	-	-	-	-	-	-	-	-
*S. purpurea*	**5.7**	-	+	-	+	-	-	-	-
*S. purpurea* x *viminalis*	+	+	-	-	-	-	-	-	+
*S. myrsinifolia*	**5.8**	-	-	4.4	-	1.1	4.0*	-	**6.5**
*S. aurita*	+	-	-	-	2.9	+	-	-	-
*S. bebbiana*	+	-	-	-	+	2.2	-	-	-
*S. caprea*	+	-	-	-	-	**3.7**	+	-	-
*S. cinerea*	3.5	+	-	-	+	-	3.4**	-	-
*S. cinerea* x *aurita*	1.6	-	-	-	+	+	-	-	-
*S. silesiaca*	-	-	-	-	+	1.5*	-	-	-
*S. lapponum*	-	-	-	-	-	-	-	1.8	-
*S. gmelinii*	-	-	+	-	-	-	-	-	-
*S. viminalis*	+	+	3.4	-	**8.7**	-	-	-	**8.6**

**Table 6.** Parameters taken from the graphs in Figure 6, showing overall similarity in gross faunistic/floristic patterning between willows and psyllids.

		**Willows**	**Psyllids**
**local similarity**	**S_(km=0)_**	0.3203	0.3897
**local diversity**	**1- S_(km=0)_**	0.6797	0.6103
**similarity decay distance**	**km_(S=0)_**	2502km	2633km
	**slope**	0.000128	0.000148
